# Preparation of Vancomycin-Loaded Aerogels Implementing Inkjet Printing and Superhydrophobic Surfaces

**DOI:** 10.3390/gels8070417

**Published:** 2022-07-04

**Authors:** Patricia Remuiñán-Pose, Clara López-Iglesias, Ana Iglesias-Mejuto, Joao F. Mano, Carlos A. García-González, M. Isabel Rial-Hermida

**Affiliations:** 1 I + D Farma Group (GI-1645), Departamento de Farmacoloxía, Farmacia e Tecnoloxía Farmacéutica, Faculty of Pharmacy, iMATUS and Health Research Institute of Santiago de Compostela (IDIS), Universidade de Santiago de Compostela, 15782 Santiago de Compostela, Spain; patricia.remuinan@rai.usc.es (P.R.-P.); clara.lopez.iglesias@rai.usc.es (C.L.-I.); ana.iglesias.mejuto@rai.usc.es (A.I.-M.); 2 CICECO Aveiro Institute of Materials, Chemistry Department, University of Aveiro, 3810-193 Aveiro, Portugal; jmano@ua.pt

**Keywords:** aerogels, chronic wounds, vancomycin, gel inkjet printing, superhydrophobic surfaces, 3D droplet printing, alginate, bioaerogels

## Abstract

Chronic wounds are physical traumas that significantly impair the quality of life of over 40 million patients worldwide. Aerogels are nanostructured dry porous materials that can act as carriers for the local delivery of bioactive compounds at the wound site. However, aerogels are usually obtained with low drug loading yields and poor particle size reproducibility and urges the implementation of novel and high-performance processing strategies. In this work, alginate aerogel particles loaded with vancomycin, an antibiotic used for the treatment of *Staphylococcus aureus* infections, were obtained through aerogel technology combined with gel inkjet printing and water-repellent surfaces. Alginate aerogel particles showed high porosity, large surface area, a well-defined spherical shape and a reproducible size (609 ± 37 μm). Aerogel formulation with vancomycin loadings of up to 33.01 ± 0.47 μg drug/mg of particle were obtained with sustained-release profiles from alginate aerogels for more than 7 days (PBS pH 7.4 medium). Overall, this novel green aerogel processing strategy allowed us to obtain nanostructured drug delivery systems with improved drug loading yields that can enhance the current antibacterial treatments for chronic wounds.

## 1. Introduction

Wounds are physical traumas where the integrity of the skin or any other tissue is compromised. The normal wound healing process has different overlapping phases, namely, hemostasis/inflammatory, proliferative and remodelling phases. A chronic wound appears when the injury is not capable of healing during those phases in a determined period of time [1,2]. Common chronic wounds include ulcers, diabetic foot ulcers, pressure ulcers, surgical wounds or infectious wounds. These injuries represent a global health problem due to the reduction in quality of life of patients, with pain, stress and usually, several work leaves [3]. From a health-economics perspective, chronic wounds could require daily health workers’ attention and possible surgical interventions. Economic forecasts expect expense growth of up to USD 27.8 billion by 2026 [4].

Chronic wounds can be prevented with suitable, effective and space- and time-accurate treatments [5]. Ideal drug release in the wound healing process must be a controlled and local release with two phases: (i) a burst release with immediate therapeutic effect at the infected site and (ii) a sustained release for a prolonged period of time [6]. Currently, there are several commercially available wound dressings, including foams, gauzes and hydrocolloids. However, they do not solve simultaneously chronic wound problems such as odour, pain, limited ability to absorb blood or exudates, control bacterial infections or promotion of cell migration and skin regeneration. Moreover, new technologies such as regenerative medicine and antimicrobial or bioactive materials are being used to develop next-generation dressings [7].

Aerogels are defined as solid, lightweight, open porous networks endowed with unique properties such as low bulk density (0.05–0.3 g/cm^3^), high porosity (>95%), very high specific surface area (>200 m^2^/g) and wide capacity of swelling (i.e., absorbent) [8]. Therefore, aerogels could be excellent materials in biomedicine because they allow fast initial biological fluid absorption and can also act as a carrier for bioactive compounds with a high loading capacity [7,9]. Aerogel-based formulations have been proposed as effective therapeutic solutions for wound treatment [7,10,11,12,13,14,15]. Compared with hydrogels (i.e., crosslinked hydrophilic polymeric networks where the internal phase is a hydrophilic solvent [16]), aerogels rely on an extraordinary degree of swelling of the dried network. They have a glove-like fitting capacity to the exudative wound morphology as well as a triggered release of their drug payload after contact with the wound fluid [13,17]. Furthermore, aerogels open the possibility of including hydrophilic and hydrophobic active agents within the matrix [18].

Biopolymer aerogels (bioaerogels) are usually preferred for these purposes because they can be obtained from sustainable sources, can have their own biological activity, and are well tolerated and non-toxic. Bioaerogels also usually have at their disposal a wide variety of available functional groups at the surface for the interaction with the biological environment and further chemical tailoring [5,19,20]. Particularly, alginate is a superabsorbent and haemostatic biopolymer, widely used in the development of drug delivery systems [16]. Moreover, alginate is able to stimulate fibroblasts, targeting growth factors and promoting granulated tissue formation [21,22]. Due to these characteristics, the use of alginate aerogels in wound healing could be suitable due to the capacity to maintain a balance between humidity, amount of exudate and gas permeation—important factors for good wound healing. Their high surface area is an advantage in absorbing a high amount of exudate and improving contact with the wound area. Furthermore, they have the simultaneous capacity of being flexible enough to fit the natural shape of wounds and of avoiding all potential sources of infection [8,19]. Particularly, aerogels in the form of small particles or beads are very interesting for reaching the application site in the case of deep chronic wounds [7].

Bioaerogels are usually prepared by extraction of the liquid phase of a hydrogel without alteration of the inner polymer structure. The most common preparation scheme involves the formation of the hydrogel precursor, followed by a solvent exchange and an extraction of the solvent. Conventional methods to obtain hydrogel particle precursors (electrostatic/vibrating nozzle, atomisation or jet cutting) are based on dripping a polymer solution on a bath containing the crosslinking agent, so that droplets are gelled in the form of spheres [23]. Despite the simplicity of these methods, droplets freely falling under gravitational forces may lead to polydisperse particles. To prepare drug-loaded hydrogel spheres, the drug can be loaded in the initial polymer solution; however, high volumes of gelation bath may lead to a prompt drug diffusion, resulting in decreased loading efficiency [24]. Finally, the use of a supercritical drying process is advantageous for the extraction step as it leads to inner mesoporous structures, differently from atmospheric and freeze-drying processes [7,23,25,26].

Gel inkjet 3D printing is an alternative method that allows the production of gel particles with narrow size distributions [27,28,29]. Gel inkjet printing is an accurate and flexible technique with a drop volume range in the micro- to picoliter range at high throughputs [27,30]. On the other hand, superhydrophobic surfaces bring new possibilities to enhance the drug entrapment, showing a virtual 100% yield since the encapsulation of the active occurs in the air–solid interface, reducing the use of solvents. These surfaces are able to repel polar dispersions of hydrophilic polymers into polar solvents. The contact angles formed between the drop and the surface are higher than 150°, so the obtained drops have a spherical shape. These surfaces have been used for the development of polymeric hydrogels with a perfectly spherical shape [31]. This technology is simple, reproducible and biocompatible and widely used in several drug delivery formulations and tissue engineering approaches [30,31,32].

Local administration of antibiotics is commonly preferred to promote wound healing and fight against possible infections, while avoiding systemic side effects and potential bacteria drug resistance [33]. There are several products in the market that have implemented a topical delivery, as they can be a good help to remove biofilm and avoid multidrug-bacteria resistance in the wound site [34]. Vancomycin is a common choice to treat *Staphylococcus aureus* infections, the most frequent Gram-positive bacteria in chronic wound diseases [10]. Vancomycin topical administration allows therapeutic levels (minimum inhibitory concentration (MIC) for *Staphylococcus aureus* of 2 μg/mL) without systemic side effects [35]. Conventional dropping methods to obtain drug-loaded aerogels (including vancomycin) demonstrated a trend of low loading efficiency, with a load percentage of vancomycin and other actives of around 7–12% [5,10,11,23].

An innovative technological combination of 3D-printing and water-repellent surfaces is herein proposed as a proof-of-concept to obtain drug-loaded aerogel microspheres at uniform particle size, shape reproducibility and expected high drug loading efficiency. To the best of our knowledge, there is not another aerogel system developed implementing the combination of both technologies. Alginate aerogels loaded with vancomycin were obtained through gel inkjet printing of an aqueous alginate solution (0.1–1.0 wt.%) into water-repellent surfaces and followed by supercritical drying. Several drug loading strategies (in the ink, bath or ink + bath) were tested. The obtained aerogels were characterised in terms of particle size by optical and scanning electron microscopies, textural properties by N_2_ adsorption–desorption analysis and drug loading yield, as well as their release profile in a simulated body fluid medium (PBS pH 7.4).

## 2. Results and Discussion

### 2.1. Definition of Operating Window for Alginate Aerogel Preparation

The classical ionic gelation mechanism of alginate has been integrated into the technological development with inkjet printing herein explored to obtain hydrogel particles of high sphericity and reproducible size. Alginate is able to form links between adjacent chains in an *egg-box* conformation [36]. Generally, these bonds are established with divalent and trivalent cations (typically Ca^2+^). These links allow us to encapsulate a broad type of bioactive agents, including, for example, monoclonal antibodies [16].

Injection pressure, nozzle-to-bath distance, output cycle time and printhead speed were crucial parameters for inkjet printing to produce spheres with uniform size on the superhydrophobic surface. High injection pressures and distances between the nozzle and the gelation bath resulted in flattened drops due to the heavy impact of solutions above the superhydrophobic surface. If the printhead speed was high and drop output cycle time (i.e., frequency of droplet ejection) was low, drops were printed within a short time of each other and agglomeration of several drops was observed [27,37,38].

**Figure 1:**
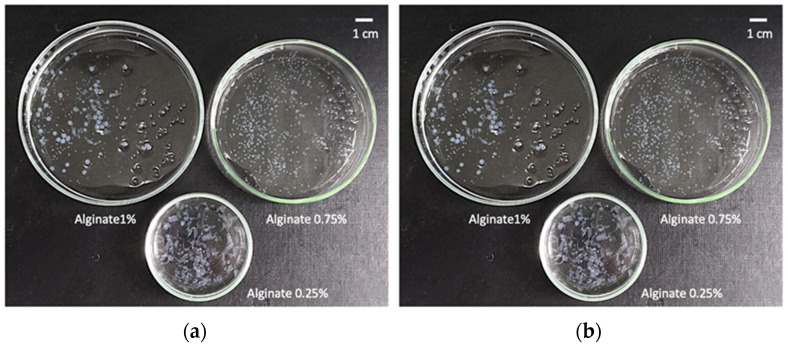
Images of gels from the different alginate concentrations tested for the search of the optimum concentration (**a**) before (hydrogels) and (**b**) after (aerogels) the scCO_2_ drying.

The optimum concentration of alginate within the aqueous ink formulations was established by visual observation of 50 drops of each alginate concentration printed using the method described in Section 4.3.1. Inks with alginate concentrations of 0.1 and 0.25% (*w/v*) resulted in very diluted hydrogels, flattened and without enough consistency for handling. Hydrogel particles obtained from inks with 0.5% (*w/v*) concentration also resulted in deformed particles, although to a lesser degree. Instead, hydrogels obtained from inks with 0.75 and 1% (*w/v*) alginate were mostly spherical and with good consistency. However, hydrogels from inks of 0.75% (*w/v*) alginate concentration hydrogels were chosen as the optimum ones (Figure 1) since the pressure used (40 kPa) was half of the value needed to be able to print drops from 1% (*w/v*) alginate inks.

Drops of 0.75% (*w/v*) alginate solution were printed and gelified using 5 mL CaCl_2_ aqueous bath solutions of different concentrations (0.2, 0.5, 0.8 and 1 M) over a superhydrophobic surface (cf. Section 4.3.2). The use of 0.2 M CaCl_2_ solutions resulted in a slow gelation process. The use of 1 M CaCl_2_ solutions resulted in a very fast gelation only able to gelify the outer shell of the sphere, resulting in hollow particles. Trade-off CaCl_2_ concentrations were set to the 0.5–0.8 M range, with 0.8 M selected for ulterior tests because of faster gelation. At a 0.8 M CaCl_2_ concentration, drops began to gelify with a transparent appearance as soon as they were in contact with the gelation bath. As gel particles aged, their appearance changed to a whitish colour that was maintained after the solvent exchange and supercritical drying steps reported in Section 4.3.3.

### 2.2. Production of Vancomycin-Loaded Aerogels Using Superhydrophobic Surfaces

Alginate aerogels were loaded with vancomycin in situ during the gelation process. Vancomycin is a very water-soluble drug and is non-soluble in ethanol. Therefore, vancomycin can be dissolved in high amounts in both the aqueous ink and in the gelation bath. Solvent exchange from the aqueous matrix of hydrogels to pure ethanol avoided additional losses during the aerogel obtaining process. The development of three types of vancomycin-loaded aerogels was intended to compare the capacities of those types to load increasing amounts of the bioactive agent.

After supercritical drying, vancomycin loading of aerogel formulations was 26.59 ± 0.17, 17.22 ± 0.35 and 33.01 ± 0.47 µg vancomycin/mg of type I, II and III particles, respectively (Table 1). These values are quite high if compared to vancomycin-loaded bioaerogels previously reported (ca. 8.5 ± 4.0, 12.9 ± 1.0 and 27.3 ± 2.8 μg vancomycin/mg of particle [10]). The results obtained correlate with the initial composition of the forming solutions. Type III aerogels, where vancomycin was initially present in the alginate ink and in the CaCl_2_ bath, were the ones with the highest loading. Type I aerogels had a lower drug loading as with this strategy; the drug is only encapsulated by impregnation when the hydrogels adsorbed the vancomycin in the gelation bath. Finally, type II aerogels were the ones with the lowest loading due to the possible diffusion of vancomycin to the crosslinking bath during the process, as the vancomycin is water-soluble. Type II and III aerogels had an encapsulation yield of 15.63 (±0.10) and 19.41 (±0.28), respectively, which are coherent yields if conventional methods were used (12.0 ± 2.0%) [5,10,11,23]. Despite the loading yields being higher than the reported ones, we hypothesise that vancomycin diffusion took place when the gels were in contact with the crosslinking bath to a certain extent. Nevertheless, the volume of this bath was 8 mL, i.e., the minimum possible at the moment, so there is room for improvement of load yields in further studies.

### 2.3. Morphological and Physicochemical Characterisation of Alginate Gels and Aerogels

**Figure 2:**
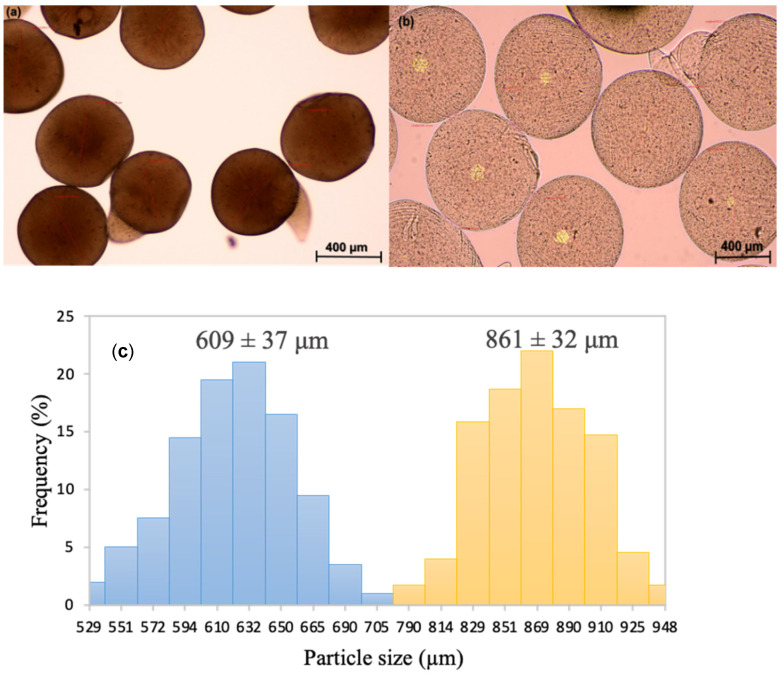
Optical images of 0.75% (*w/v*) alginate (crosslinked in 0.8 M CaCl_2_ baths) (**a**) aerogels and (**b**) hydrogels obtained by inkjet printing, with (**c**) the particle size distribution (in number) of aerogels (blue) and hydrogels (yellow), respectively.

Aerogel and hydrogel Feret diameters were measured using an optical microscope to determine the particle size distribution and the volume shrinkage taking place upon aerogel processing. The average diameter of the hydrogels was 861 ± 32 µm, while the average diameter of the aerogels was 609 ± 37 µm (Figure 2). The standard deviation of the aerogels was very low; consequently, they can be considered uniform due to their narrow size distribution compared with traditional methods of gel preparation [23]. The degree of the volume shrinkage was 64.61%, a similar value compared with literature (57.0 ± 5.0%) [10]. Moreover, aerogel circularity was 80.2 ± 0.7%, while hydrogel circularity was 73.7 ± 0.6%.

**Figure 3:**
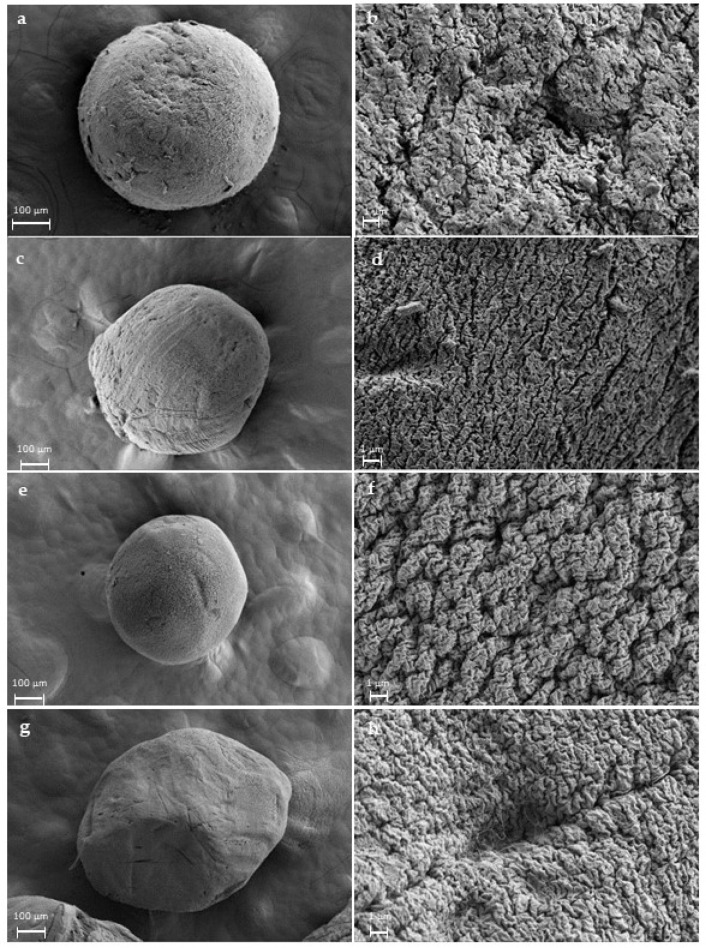
Blank 0.75% (*w/v*) alginate aerogels (**a**,**b**); type I aerogels (**c**,**d**); type II aerogels (**e**,**f**); type III aerogel (**g**,**h**) structures. In higher magnification images, porous structure is observed in all formulations.

Aerogel surface morphology and inner structure were analysed by SEM for both blank and loaded ones (Figure 3). A well-defined spherical shape was shown in all formulations. Moreover, the characteristic porous structure with the strongly interconnected fibrous network of alginate aerogels was also observed. These fibres are mixed with each other, and the generated voids are responsible for the porosity both in the outer and inner aerogel structure. The presence of mesoporosity was confirmed by nitrogen adsorption-desorption analysis (Table 2, Figures S1 and S2), with typical parameters for these systems [39,40]. The amino groups of vancomycin confer the ability to bond via electrostatic interactions to carboxyl groups of alginate of the ink [41]. This could be an explanation for this effect of vancomycin in the textural properties, as the said drug may act as an extra crosslinking agent, which would stabilise the structure and better prevent shrinkage during the solvent exchange and supercritical drying processes.

Aerogels evaluated by nitrogen adsorption–desorption showed type IV isotherms according to IUPAC recommendations (Figure S1). The isotherm morphology from nitrogen adsorption–desorption tests is typical of mesoporous materials and is characterised by a huge volume of nitrogen molecules adsorbed at high relative pressures [42]. Physisorption studies using non-reactive molecules, like nitrogen adsorption–desorption tests, are usually preferred for textural analysis over other chemisorption tests (such as ammonia or deuterated water) as they do not react with the functional groups of the polymer chains [43]. In the initial part of the isotherm, the adsorption of the monolayer was completed, and then multilayer adsorption started. An H1 hysteresis loop was observed, which is associated with capillary condensation in the mesopores. Aerogels also presented a log-normal, unimodal pore size distribution according to the BJH method. The surface properties of biopolymer aerogels have a great influence on biological processes such as cell attachment and proliferation, protection against bacteria and fluid sorption capacity [7].

**Figure 4:**
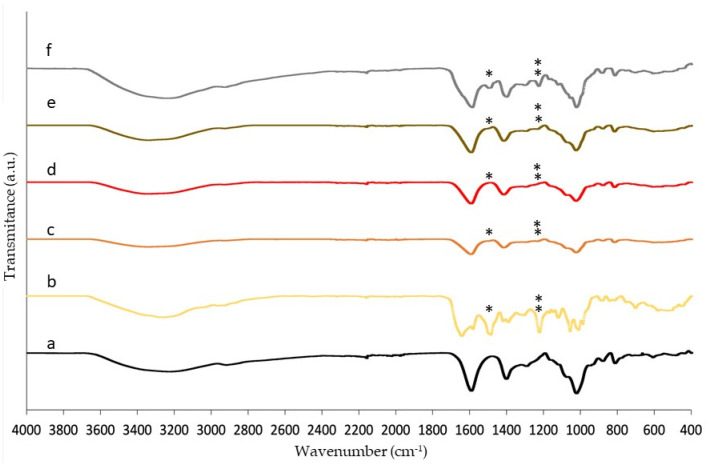
FTIR spectra of (**a**) alginate blank aerogels; (**b**) vancomycin; printed aerogels; (**c**) type I: 0.75% alginate in the ink and a saturated bath of vancomycin in CaCl_2_ O.8 M; (**d**) type II: 0.75% alginate and 17% (*w/w* alginate) vancomycin forming part of the ink; (**e**) type III: drug both in the saturated vancomycin aqueous bath and forming part of the ink and (**f**) the physical mixture of alginate and vancomycin powders. Single asterisk and double asterisks indicate the presence in all formulations of typical bands of vancomycin at 1540 cm^−1^ and 1230 cm^−1^ respectively.

FTIR results show the presence of the vancomycin within the structure of the aerogels compared with the blank ones (Figure 4). The absorption bands of the vancomycin at 3450, 1654, 1504 and 1230 cm^−1^ for hydroxyl stretching, C=O stretching, C=C and phenols were observed in the vancomycin spectrum [10]. However, due to overlapping with the alginate bands, very slight changes in the formulations were observed compared with the spectrum of the polysaccharide at ca. 1540 cm^−1^ (single asterisk, Figure 4) and 1230 cm^−1^ (double asterisk, Figure 4). However, in the physical mixture of alginate and vancomycin (Figure 4f), the peaks at these wavenumbers were clearer. Since the physical mixture was evaluated at the same ratio of alginate/vancomycin as the ink, this difference in band intensities could be explained by the loss of vancomycin in the aerogel processing during the crosslinking in the gelation bath by means of diffusion.

### 2.4. Vancomycin Release Studies from Alginate Aerogels

**Figure 5:**
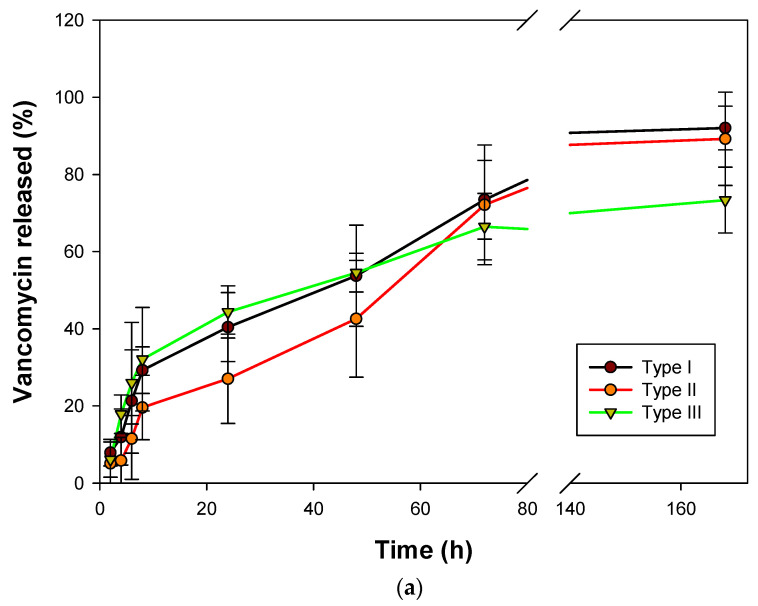

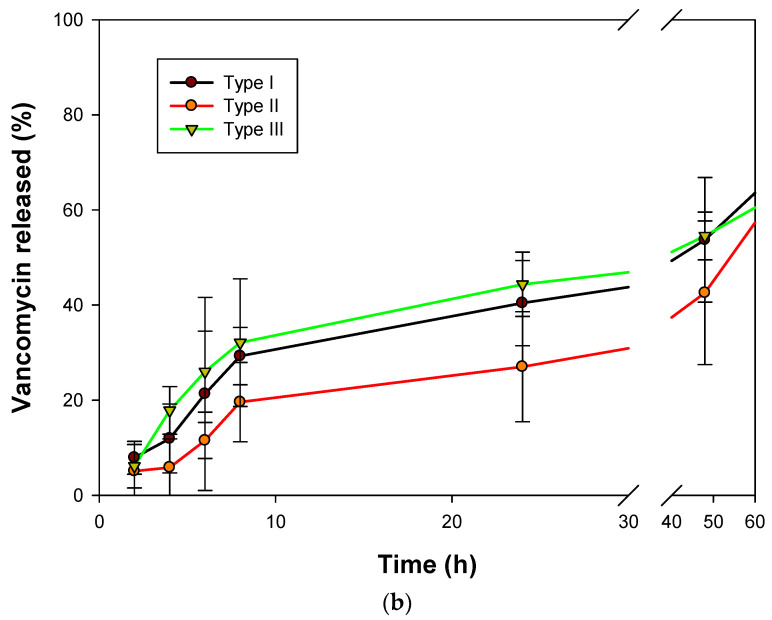
(**a**) Vancomycin released (%) from the 3 aerogel formulations in PBS pH 7.4 at 37 °C for 7 days and (**b**) magnification of the release profile in the first hours, showing the burst release and the initial phase of the plateau region.

Drug release profile from the aerogel matrices will depend on several factors such as drug and aerogel material hydrophilicity, drug crystallinity, degree of gel crosslinking, drug mass transport mechanisms, specific interactions between the drug and the aerogel, pH and temperature, among others [9]. The three types of vancomycin-loaded aerogels processed using superhydrophobic surfaces had a similar release profile with three stages. Type III aerogels provided a sustained release during a longer release period with respect to the other aerogel formulations (Figure 5).

Several release kinetics models intended for spherical drug delivery systems with swelling matrices were used to fit the vancomycin release profiles (Table 3). The Ritger–Peppas equation (Equation (1)) was the kinetic model that better characterised the release behaviour, revealing a classical kinetic release for swelling matrices:

Mt/M∞ = k·t^n^ (1)

where Mt/M∞ is the fraction (in %) of drug released at a certain time t (in h.), k is the release kinetic coefficient (h^−1^) and n is the diffusion coefficient [44].

This type of release kinetics has three typical stages; the first one is a burst release, in this case, during the first 8 h. This could be attributed to the fraction of drug in the external surface of the aerogels, which is weakly bound to the structure and also, it is easy to diffuse due to the absence of resistance of the polymeric matrix. In this release stage, there is a slower drug release from type II aerogels that can be related to the effect that the vancomycin loading has on the textural properties of the aerogels. As the surface area is higher in the case of type I and III aerogels, a faster initial release can be expected. In the case of the type II aerogels, the surface area, as well as the pore size, is lower, and thus a slower release was obtained. Then, there is a sustained release from 8 to 72 h because of a combination of Fickian diffusion and the slow erosion of the polymeric matrix. As time progressed, the matrix changed with large sizes of pores in the inner region due to the erosion of the polymeric matrix. Then, this erosion controls the release with a slow drug release ratio [45,46].

In the Ritger–Peppas model, the governing mass transport mechanism of the drug from the carrier system to the release medium depends on the geometry and structure of the said carrier (Figure S3). In spherical-shape systems, the value of the diffusion exponent (n) indicates the drug release mechanism. As the n-value of aerogels type I is in the range of 0.43 to 0.85, it could be classified as anomalous transport, thus non-Fickian diffusion. In the case of type II and III aerogels, the mechanism could be assumed as Fickian diffusion. [44]

The minimum inhibitory concentration (MIC) for sensitive bacterial strains *Staphylococcus aureus* (2 µg/mL) was outperformed in all drug release tests after the first 2 h so that the aerogels exhibited initially rapid and subsequently sustained antimicrobial activity over time [10]. The test was performed for 7 days due to the intended biomedical application.

## 3. Conclusions

For the first time, vancomycin-loaded alginate aerogels were successfully developed using a novel 3D printing method implementing superhydrophobic surfaces. The use of this class of surfaces is an efficient method to obtain spherical and uniform size aerogel particles with very low variability (aerogel circularity was 80.2 ± 0.7%). This could lead to the preparation of more efficient drug-loaded aerogels to deliver the agent due to the increase of the specific surface area and the mesoporosity achieved. Furthermore, the implementation of the combination of 3D printing and superhydrophobic surfaces is versatile in terms of the use of different methods for the preparation of drug-loaded aerogels, as is proved in this work with vancomycin loading. All the loading strategies used herein were able to deliver the bioactive agent in PBS pH 7.4 medium for 1 week. The quantity of drug released from the alginate aerogels is enough to treat the most common infection in chronic wounds, reaching therapeutic levels. The possibility of a theoretical 100% encapsulation yield in the hydrogel formation could lead to aerogels with an improvement in the drug payload and will be the subject of future studies. Further research will also focus on the evaluation of the antimicrobial efficiency of the obtained aerogels and the evaluation of the versatility of this technological combination for the incorporation of different wound healing promoting agents.

## 4. Materials and methods

### 4.1. Materials

Alginic acid sodium salt from brown algae (guluronic/mannuronic acid ratio of 70/30) was supplied by Sigma Life Science (Irvine, UK). Vancomycin hydrochloride (C_66_H_75_Cl_2_N_9_O_24_·HCl, 94.3% purity) was supplied by Guinama (Valencia, Spain). Calcium chloride anhydrous (CaCl_2_, >99% purity) was supplied by Scharlab (Barcelona, Spain). Absolute ethanol (EtOH, >99.9% purity) and CO_2_ (99.8% purity) were purchased from VWR Chemicals (Fontenay-sous-Bois, France) and Nippon Gases (Madrid, Spain), respectively. Tetraethylorthosilicate (TEOS, 98% purity), ammonium aqueous solution 30–33% and 1H,1H,2H,2H-perfluorodecyltriethoxisilane (PFDTS, 97% purity) were from Sigma–Aldrich (Darmstadt, Germany). Water was purified using reverse osmosis (resistivity > 18 MΩ·cm, Milli-Q, Millipore^®^, Madrid, Spain).

Ultrapure nitrogen (N_2_ (g), >99% purity) supplied by Praxair (Madrid, Spain) was used for the adsorption-desorption textural analysis. Phosphate buffered saline (PBS) pH 7.4, and potassium dihydrogen phosphate (KH_2_PO_4,_ 98.0–100.5% purity) were both supplied by ITW Reagents (Barcelona, Spain). Sodium hydroxide (NaOH, 99% purity) and acetonitrile (CH_3_CN, ≥99.9% purity) were both purchased from VWR Chemicals (Barcelona, Spain).

### 4.2. Preparation of Superhydrophobic Surfaces

The production of polystyrene superhydrophobic surfaces was performed using a simple, economical and fast procedure based on the protocol previously described [47]. In brief, polystyrene Petri dish plates were spray-coated with UV-resistant FluoroThane-MW reagent (WX 2100™) that provides a contact angle of about 150°, as described by the manufacturer (Cytonix, Beltsville, MD, USA). The Petri dish surface was spray-coated and left to dry overnight in a chemical safety fume hood at room temperature. On the following day, the surface was washed with ethanol (99%) and oven dried at 37 °C for 5 days.

### 4.3. Preparation of Alginate Aerogel Microspheres

#### 4.3.1. Determination of Optimal Concentration of The Alginate and CaCl_2_ Solutions

Alginate hydrogel particles were prepared following the sol-gel method and consequently crosslinked with an ionic crosslinker (CaCl_2_). Alginate solutions with concentrations of 0.1, 0.25, 0.5, 0.75 and 1.0% (*w/v*) were used as inks and loaded into the 6 mL cartridge of the inkjet printhead of BIO X^TM^ printer (Cellink, Gothenburg, Sweden) to evaluate their printability. Drops were printed in a Petri dish at room temperature to analyse them visually. Printing parameters, such as printhead speed (8–20 mm/s), injection pressure (10–55 kPa), drop output cycle time (250–450 ms), microvalve opening time (5–15 ms) and pattern density (1.5–3.5%), were modified for each alginate concentration.

Drops with the optimum alginate concentration (0.75% (*w/v*)) were inkjet printed on a superhydrophobic surface and crosslinked in gelation baths of CaCl_2_ at different concentrations (0.2, 0.5, 0.8 and 1 M). Gelation time of alginate drops was evaluated. Optimum concentrations of the alginate and CaCl_2_ solutions were chosen, taking into account the best printing parameters obtained among the different concentrations of the alginate and CaCl_2_ tested in our laboratory_._

#### 4.3.2. Preparation of Alginate Hydrogels by Gel Inkjet Printing Using Superhydrophobic Surfaces

Three millilitres of 0.75 % (*w/v*) alginate concentration were loaded into the printing cartridge. The superhydrophobic surface was placed at a slant, and 8 mL of 0.8 M CaCl_2_ was added at the bottom of it. Drops were printed from the top of the surface and rolled along it (ca. 8 cm) until the gelation bath. Optimum printing parameters were 16 mm/s printhead speed, 40 kPa pressure, 300 ms drop output cycle time, 10 ms microvalve opening time and 3.5% pattern density. Grid patterns were 30 mm × 30 mm × 1 mm (ca. 65 drops). Alginate beads were left in the gelation bath for 24 h at room temperature and pressure. These parameters were experimentally determined. (Video S1).

**Figure 6:**
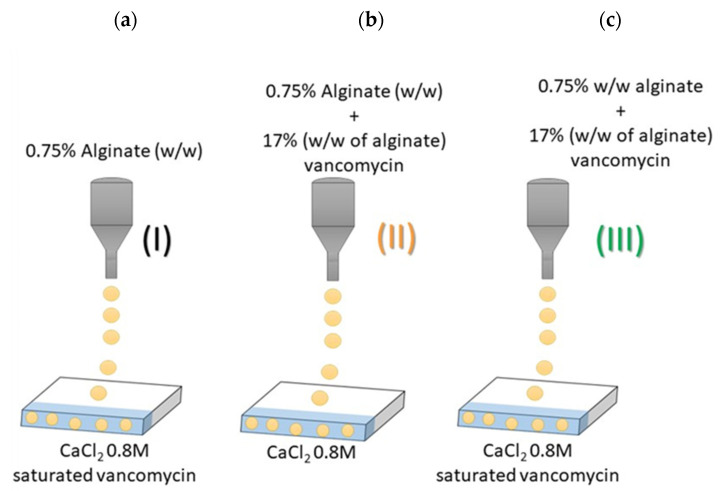
Different ink and gelation bath compositions for inkjet-printing of hydrogels; (**a**) Type I: 0.75% (*w/v*) alginate in the ink and a saturated bath of vancomycin in CaCl_2_ O.8 M; (**b**) Type II: 0.75% (*w/v*) alginate and 17% (*w/w* alginate) vancomycin forming part of the ink; and (**c**) Type III: drug both in the saturated vancomycin bath and being part of the ink.

Three different printing strategies were evaluated to produce vancomycin-loaded hydrogels. In the first type of formulation (Type I), vancomycin at a saturated concentration (25 mg/mL) was placed in the 0.8 M CaCl_2_ bath. In type II, 17.0 % (*w/w*) vancomycin was included within the alginate ink. The third type (Type III) of sample had vancomycin in the ink and in the CaCl_2_ bath at the same concentration as in formulations I and II (Figure 6).

#### 4.3.3. Solvent Exchange and Supercritical Drying of Alginate Gels

Ninety millilitres of each vancomycin-loaded gel type (Section 4.3.2.) were produced. Then, two sequential solvent exchanges of the alginate gels with absolute EtOH were carried out at a frequency of 24 h to eliminate water from the gel particles. Alcogel particles were introduced into paper cartridges and put into a 100 mL autoclave (TharSFC, Pittsburg, PA, USA). Twenty millilitres of EtOH were previously added to avoid the premature evaporation of the EtOH contained in alcogels. During the drying process (3.5 h), temperature and pressure were 40 °C and 120 bar, respectively, with a CO_2_ flow of 5–7 g/min passing through the autoclave. Ethanol extracts were taken out and weighed at selected drying times to monitor the supercritical process. Finally, the equipment was depressurised, and the aerogels were collected from the autoclave for further characterisation [10].

### 4.4. Alginate Gels Characterization

#### 4.4.1. Morphological and Physicochemical Properties of Alginate Beads

Morphological and physicochemical characteristics were analysed similarly to other aerogel formulations [14,48]. Firstly, hydrogel and aerogel particle diameters were determined by using a CKC53 optical microscope equipped with an EP50 camera and using EPview image analysis software v.1.3 (Olympus, Tokyo, Japan). Surface structure of aerogel microspheres was studied by scanning electron microscopy (SEM) using a FESEM Ultra-Plus microscope (Zeiss, Jena, Germany). Aerogels were previously sputter-coated with a 10 nm layer of iridium to improve the contrast (Q150 T S/E/ES equipment, Quorum Technologies, Lewes, UK). Then, textural properties of aerogel particles were characterised by nitrogen adsorption–desorption analysis (ASAP 2000, Micromeritics, Norcross, GA, USA). Samples were degassed under vacuum at 40 °C for 24 h. The Brunauer–Emmet–Teller (BET) and Barrett–Joyner–Halenda (BJH) methods were applied to determine the specific surface area (a_BET_), pore size distribution, pore diameter (Dp) and pore volume (Vp). Lastly, attenuated total reflectance/Fourier-transform infrared spectroscopy (ATR/FT-IR) was performed with a Gladi-ATR accessory using a diamond crystal (Pike, Madison, WI, USA). Raw vancomycin, blank alginate aerogels, a physical mixture and the three formulations in the powdered form were characterised in the 400–4000 cm^−1^ IR-spectrum range using 32 scans at a resolution of 2 cm^−1^.

#### 4.4.2. Vancomycin Drug Content and Loading Efficiency

Vancomycin entrapment yield of the different aerogel formulations was evaluated with ca. 20 mg of each sample placed in Eppendorf tubes with 5 mL of PBS pH 7.4 buffer solution. Tests were carried out in triplicate. Samples were introduced in ultrasound equipment (Branson Ultrasonics, Danbury, CT, USA) for 30 min to completely dissolve the drug. Then, 1 mL of sample was filtered (PTFE hydrophilic, 13 mm, 0.22 μm) and introduced into HPLC glass vials. Jasco LC-4000 HPLC (Madrid, Spain) equipped with a C_18_ column (symmetry columns, 5 µm, 3.9 × 150 mm) was used to measure the drug content. HPLC method conditions were set at 25 °C, a mobile phase of phosphate buffer (30 mM, pH 2.2) and acetonitrile (86:14% *v/v*) operating at an isocratic flow of 0.72 mL/min for 7 min. Chromatograms were obtained at the wavelength of 205 nm [49]. Previously, a calibration curve of vancomycin was obtained and validated in the 0.5–50 μg/mL range (R^2^ > 0.999) [50].

#### 4.4.3. Vancomycin Release from Alginate Aerogels

Vertical Franz diffusion cells with a 6.2 mL volume in the receptor compartment fitted with cellulose nitrate filters (pore size 0.45 μm) as membranes were employed in the test. Tests were carried out in quadruplicate at 37 °C and 70 rpm of continuous stirring with Heidolph Incubator 1000 equipment (Schwabach, Germany). Franz diffusion cells were filled with PBS buffer pH 7.4 as a release medium, and ca. 20 mg of aerogels were placed into the cells. Then, 200 μL of PBS was added to the donor compartment containing the aerogels to mimic the wet wound environment (time 0). Aliquots of 0.9 mL of release medium were taken at pre-established times (2, 4, 6, 8, 24, 48, 72, 96 and 168 h) in the receptor chamber, and vancomycin content was monitored by HPLC. The extracted volume was immediately replaced with equal volumes of fresh PBS medium. Vancomycin content was measured using the same HPLC method reported in Section 4.4.2.

For the kinetic fitting, the following equations were applied [10,51]:

Zero order (Equation (2)):

Mt/M∞= kt(2)

where Mt/M∞ is the amount of vancomycin released (%) at time t, k the release kinetic coefficient and t the time in h.

First order (Equation (3)):

Mt/M∞= 1+ e^−k^_1_^t^ (3)

where Mt/M∞ is the amount of drug released (%) at time t, k_1_ is the release kinetic coefficient and t, the time in h.

Higuchi model (Equation (4)):

Mt/M∞ = k_2_ t ^½^ (4)

where Mt/M∞ is the amount of drug released (%) at time t, k_2_ is the release kinetic coefficient and t, the time in h.

## Figures and Tables

**Table 1:** Vancomycin loading and entrapment yield of different aerogels using superhydrophobic surfaces.

	Type I	Type II	Type III
Vancomycin loading (µg/mg particles)	26.59 (±0.17)	17.22 (±0.35)	33.01 (±0.47)
Entrapment yield (%)	15.63 (±0.10)	10.13 (±0.20)	19.41 (±0.28)

**Table 2:** Textural properties of blank and vancomycin-loaded alginate aerogel microspheres evaluated by nitrogen adsorption–desorption tests. In parenthesis, standard deviation values.

Formulations	a_BET_ (m^2^/g) *	V_P,BJH_ (cm^3^/g) **	D_P,BJH_ (nm) ***
Blank aerogels	312 (16)	0.80 (0.04)	11.2 (0.6)
Type I	742 (37)	3.90 (0.20)	21.8 (1.1)
Type II	268 (13)	2.54 (0.13)	35.2 (1.8)
Type III	530 (26)	2.65 (0.13)	21.0 (1.0)

* Specific surface area by the BET method; ** Overall specific pore volume obtained by the BJH-method from the desorption curve; *** Mean pore diameter by the BJH-method from the desorption curve.

**Table 3:** Kinetic fitting parameters of the vancomycin release from drug-loaded alginate aerogels in PBS solution (pH 7.4) to different kinetic models. Bold letters highlight the more adequate parameters.

Kinetic Model	Parameters	Type I	Type II	Type III
Ritger-Peppas	k (h^−1^)	8.24	13.88	3.35
	n	0.48	0.39	0.41
	R^2^	0.96	0.94	0.96
Higuchi	k (h^−1/2^)	0.38	11.18	8.85
	n	8.13	8.91	6.82
	R^2^	0.95	0.93	0.97
Zero order	k (h^−1^)	13.42	6.30	22.93
	n	0.914	0.87	0.64
	R^2^	0.87	0.96	0.92
First order	k (h^−1^)	87.35	100.48	79.04
	n	0.011	0.016	0.012
	R^2^	0.93	0.93	0.97

## Data Availability

Not applicable.

## Supplementary Materials

The following supporting information can be downloaded at: https://www.mdpi.com/article/10.3390/gels8070417/s1, Figure S1: Nitrogen adsorption–desorption isotherm of blank alginate aerogels. This isotherm is representative of all aerogel formulations; Figure S2: Pore size distribution of blank and drug-loaded aerogel formulations obtained by a technological combination of inkjet printing and superhydrophobic surfaces; Figure S3: Vancomycin release kinetics fitting to Ritger-Peppas equation from alginate aerogel matrices. Dotted lines representing theorical values estimated with the model. Standard deviations are not shown for the sake of clarity; Video S1: Manufacturing of the drop-by-demand aerogels implementing superhydrophobic surfaces.
