# Supplementary material for: Preparation of Vancomycin-Loaded Aerogels Implementing Inkjet Printing and Superhydrophobic Surfaces
Source: Gels. 2022 Jul 4;8(7):417. doi: 10.3390/gels8070417 (PMC9319758; doi:10.3390/gels8070417)
Supplement: Supplementary file 1 [file gels-08-00417-s001.zip › gels-1774693-supplementary_conversion.pdf]

gels

MDPI

Article

Preparation of Vancomycin-Loaded Aerogels Implementing Inkjet Printing and Superhydrophobic Surfaces

Patricia Remuiñán-Pose [1], Clara López-Iglesias [1]; Ana Iglesias-Mejuto [1]; Joao F. Mano [2], Carlos A. García-González [1,*] and M. Isabel Rial-Hermida [1,*]

[1] Departamento de Farmacoloxía, Farmacia e Tecnoloxía Farmacéutica, I+D Farma Group (GI-1645), Faculty of Pharmacy, iMATUS and Health Research Institute of Santiago de Compostela (IDIS), Universidade de Santiago de Compostela, 15782 Santiago de Compostela, Spain; patricia.remuinan@rai.usc.es (P.R.-P.); clara.lopez.iglesias@rai.usc.es (C.L.-I.); ana.iglesias.mejuto@rai.usc.es (A.I.-M.)

[2] CICECO Aveiro Institute of Materials, Chemistry Department, University of Aveiro, 3810-193 Aveiro, Portugal; jmano@ua.pt

* Correspondence: carlos.garcia@usc.es (C.A.G.-G.); mariaisabel.rial@usc.es (M.I.R.-H.) Tel: +34881815252

Supplementary Materials

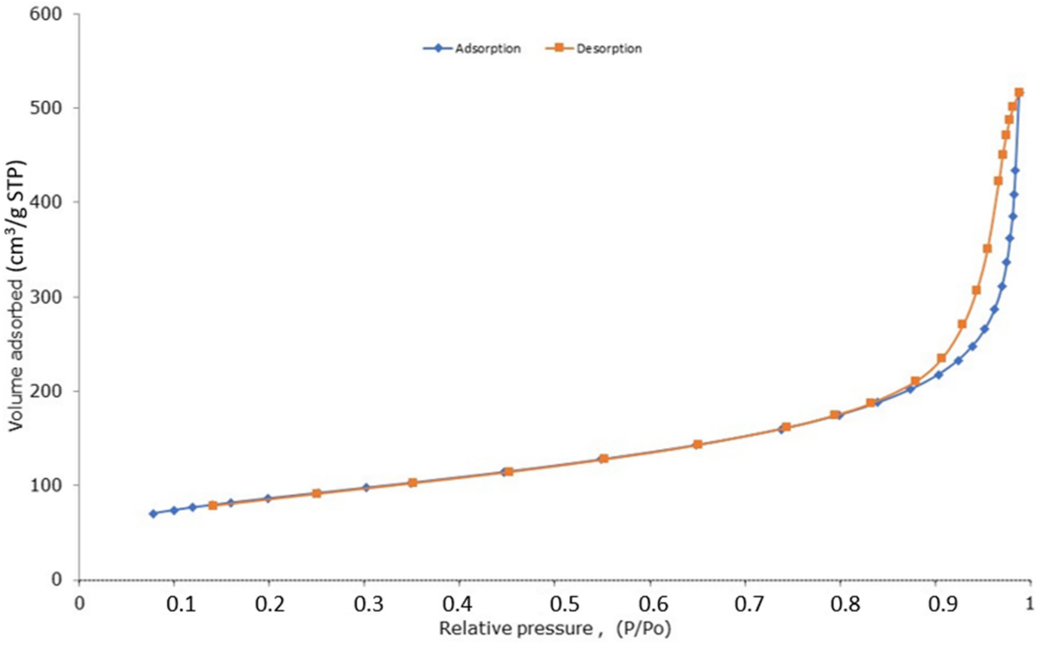

Figure S1. Nitrogen adsorption-desorption isotherm of blank alginate aerogels. This isotherm is representative of all aerogel formulations.

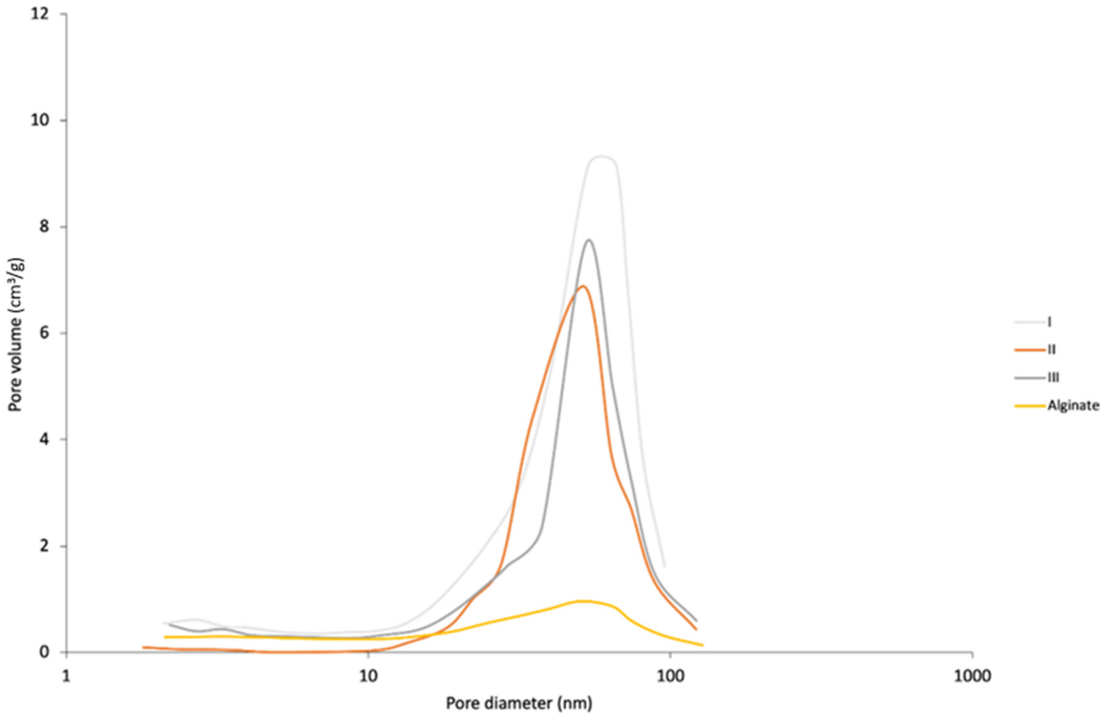

Figure S2. Pore size distribution of blank and drug-loaded aerogel formulations obtained by technological combination of inkjet printing and superhydrophobic surfaces.

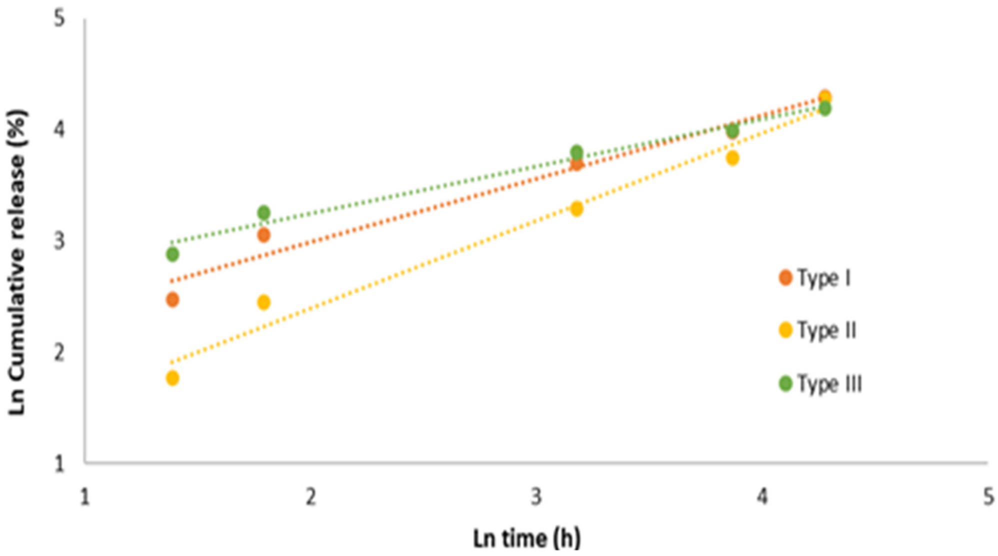

Figure S3. Vancomycin release kinetics fitting to Ritger-Peppas equation from alginate aerogel matrices. Dotted lines representing theorical values estimated with the model. Standard deviations are not shown for the sake of clarity.

Video S1: Manufacturing of the drop-by-demand aerogels implementing superhydrophobic surfaces.
